# Experimental demonstration of high fidelity entanglement distribution over decoherence channels via qubit transduction

**DOI:** 10.1038/srep15384

**Published:** 2015-10-21

**Authors:** Hyang-Tag Lim, Kang-Hee Hong, Yoon-Ho Kim

**Affiliations:** 1 Department of Physics, Pohang University of Science and Technology (POSTECH), Pohang, 790-784, Korea

## Abstract

Quantum coherence and entanglement, which are essential resources for quantum information, are often degraded and lost due to decoherence. Here, we report a proof-of-principle experimental demonstration of high fidelity entanglement distribution over decoherence channels via qubit transduction. By unitarily switching the initial qubit encoding to another, which is insensitive to particular forms of decoherence, we have demonstrated that it is possible to avoid the effect of decoherence completely. In particular, we demonstrate high-fidelity distribution of photonic polarization entanglement over quantum channels with two types of decoherence, amplitude damping and polarization-mode dispersion, via qubit transduction between polarization qubits and dual-rail qubits. These results represent a significant breakthrough in quantum communication over decoherence channels as the protocol is input-state independent, requires no ancillary photons and symmetries, and has near-unity success probability.

Beyond the fundamental interest, entanglement is considered as a key resource in quantum computing and communication tasks. The system-environment interaction, which is often unavoidable, can cause decoherence of the quantum system, resulting in degradation or complete loss of entanglement^1^. Thus, finding a way to execute quantum information protocols in the presence of decoherence is a main challenge in experimental quantum information research.

A number of schemes have been discovered to date to cope with decoherence^2^,^3^,^4^,^5^,^6^,^7^,^8^,^9^,^10^,^11^,^12^,^13^,^14^. In the decoherence-free subspace (DFS) approach^2^,^3^,^4^,^5^,^6^,^7^, the interaction Hamiltonian must satisfy particular conditions and only quantum states encoded in DFS are immune to decoherence. While there exist methods for state-independent encoding onto DFS^8^, such schemes require ancillary qubits and the theoretical success probability is less than unity. The quantum Zeno effect can also be used for state-independent entanglement protection against decoherence but in order to decrease the disentanglement rate, the period of sequential measurements should be short and each measurement reduces the final success probability^9^. Recently, it has also been shown that the weak measurement and quantum measurement reversal can effectively protect entanglement from decoherence^10^,^11^,^12^,^13^,^14^, albeit with the success probability inversely proportional to the amount of final entanglement.

Different decoherence mechanisms, however, affect qubits differently. For instance, in the case of photonic qubits, polarization-mode dispersion causes decoherence for a polarization qubit while without causing decoherence for a time-bin qubit^15^. Thus, with qubit transduction, coherent and unitary switching between different qubits (e.g., qubit encoding bases, choices of physical systems for qubits, etc), it will be possible to avoid decoherence altogether and preserve entanglement even in the presence of system-environment interaction. The term transducer typically refers to a sensing or a signal-processing device that converts a physical quantity into an electrical signal or vice versa, e.g., a microphone or a speaker. Then, a quantum transducer is a device that converts a quantum state into another while maintaining the initial quantum characteristics. To date, quantum transduction for photons typically involve frequency-upconversion for easier detection, e.g., frequency-conversion of single-photon polarization qubit^16^, frequency-conversion of telecom-band single-photons^17^, etc. Recently, quantum transduction schemes involving electromechanical and optomechanical systems have also been proposed^18^,^19^.

In this Letter, we propose and experimentally demonstrate a scheme to distribute two-qubit entanglement over decoherence channels via qubit transduction (QT). As a proof-of-principle demonstration of our scheme, we experimentally show that a two-qubit polarization entanglement can be distributed over quantum channels with two types of decoherence, amplitude damping and polarization-mode dispersion, via qubit transduction between polarization qubits and dual-rail qubits. Our qubit transduction protocol for entanglement distribution over decoherence channels is input-state-independent, requires no ancillary photons and symmetries, and has near-unity success probability.

## Results

**Figure 1:**
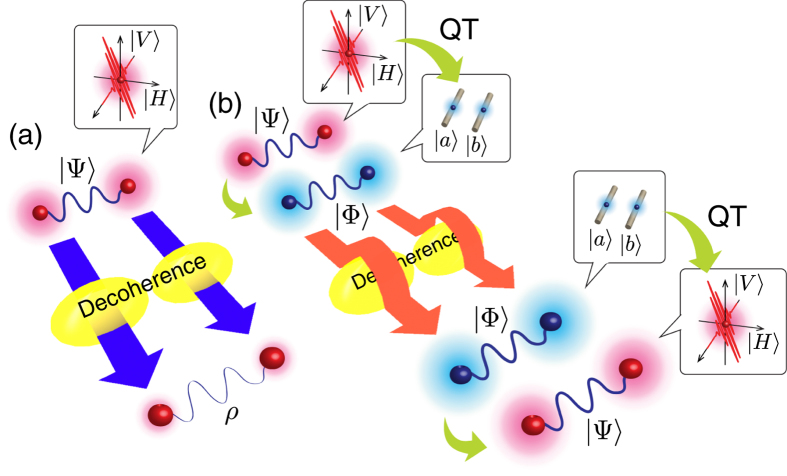
Entanglement distribution over decoherence via qubit transduction. (**a**) Alice sends a pair of polarization-entangled qubits to Bob via quantum channels with polarization-dependent decoherence. Bob receives a mixed two-qubit state with less entanglement or even zero entanglement. (**b**) Alice first performs QT, converting the polarization qubits to dual-rail qubits, before launching them over the decoherence channels. Bob performs reverse QT, reverting the dual-rail qubits back to polarization-qubits. Note that the decoherence is not assumed to be collective.

The protocol for entanglement distribution via qubit transduction is schematically shown in Fig. 1. Alice prepares a two-qubit polarization-entangled state 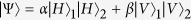, where 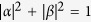 and 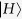 and 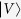, respectively, refer to the horizontal and vertical single-photon polarization states. Subscripts 1 and 2 refer to qubit 1 and 2, respectively. Alice then sends the qubits to Bob over the decoherence quantum channels as shown in Fig. 1(a). The quantum channels are subject to decoherence in the photonic polarization modes and we consider two specific types of decoherence: amplitude-damping and polarization-mode dispersion. Due to decoherence in the quantum channels, Bob receives a two-qubit state in the mixed state with less entanglement or even zero entanglement.

To avoid the effects of decoherence, Alice first performs qubit transduction, unitarily converting the polarization qubits to dual-rail qubits, before launching them over the decoherence channels, see Fig. 1(b). The initial polarization two-qubit state 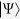 is then converted to the dual-rail two-qubit state 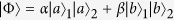, where 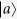 and 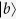 refer to the dual-rail path modes of a single-photon. As the single-photons in the dual-rail encoding have the same polarization state, the dual-rail qubits are insensitive to decoherence in the polarization modes, passing through the decoherence channels without loss of entanglement. Bob performs reverse quantum transduction to revert the dual-rail qubits back to the polarization-qubits.

Note that, unlike some experiments in DFS, the decoherence is not assumed to be collective in our protocol. As our protocol does not depend on DFS, our scheme is inherently state-independent with no requirement for ancillary qubits. Furthermore, if qubit transduction is lossless, the success probability of the protocol is unity. As we show in our work, experimental QT between polarization qubits and dual-rail qubits can be accomplished linear optically without any loss^20^,^21^. QT between polarization qubits and time-bin qubits can also be accomplished with linear optical elements and high speed modulators with very low loss^22^,^23^.

**Figure 2:**
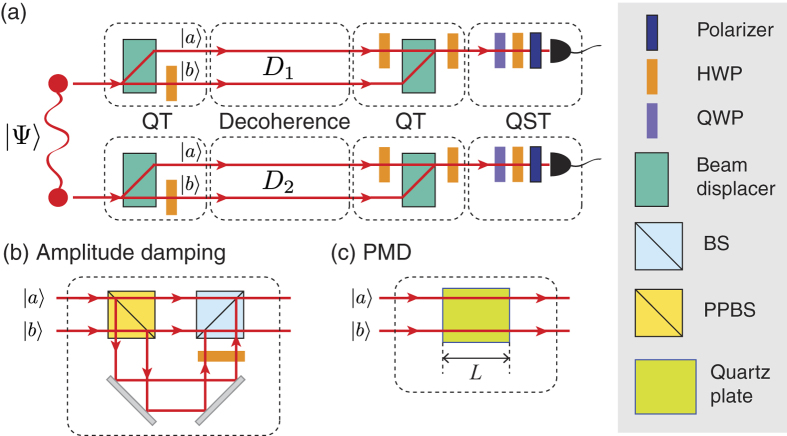
Experimental setup. (**a**) Qubit transduction (QT) is performed before an initial two-qubit polarization-entangled state is launched to the decoherence channels (*D*_1_ and *D*_2_). The QT setup consists of a polarization-dependent beam displacer (BD) and a 45°-oriented half-wave plate (HWP). After the decoherence channels, QT is performed again to convert the two-qubit dual-rail state back to a two-qubit polarization state. Quantum state tomography (QST) is performed with a half-wave plate (HWP), a quarter-wave plate (QWP), and a polarizer, to analyse the two-qubit polarization state. (**b**) Amplitude-damping decoherence is realized by using a set of a partially-polarizing beam splitter (PPBS), a beam splitter (BS), and a half-wave plate (HWP). (**c**) Decoherence due to polarization-mode dispersion (PMD) is implemented by a quartz plate of thickness *L*.

The experimental setup is schematically shown in Fig. 2 and is composed of three main parts: qubit transduction, decoherence, and quantum state tomography. Detailed information about the experimental setup and types of decoherence we considered is described in the Methods section.

**Figure 3:**
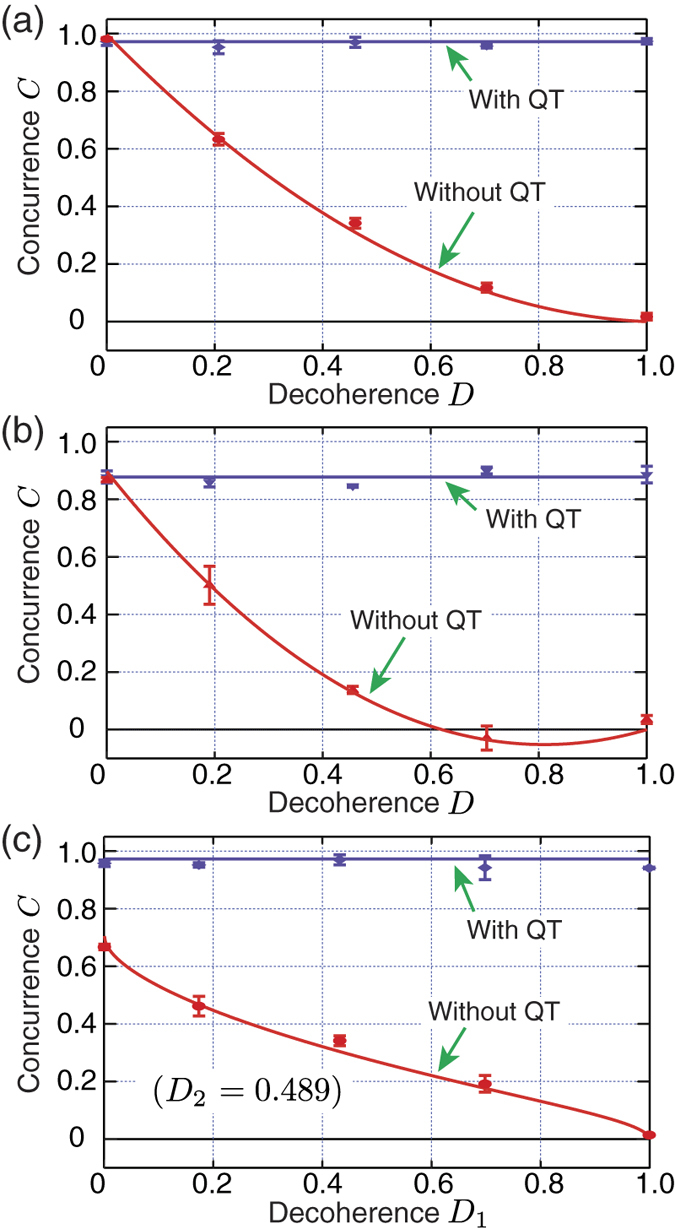
Experimental results: amplitude damping decoherence. In (**a**,**b**), both qubits experience identical decoherence *D*. For (**a**) 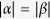 and for (**b**) 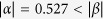. In (**c**), 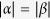 and qubit 1 experiences decoherence *D*_1_ while qubit 2 experiences a fixed decoherence *D*_2_ = 0.489. It is clear that, without QT, decoherence causes significant degradation of entanglement, even entanglement sudden death in (**b**). With QT, however, decoherence has no effect on concurrence, i.e., the amount of two-qubit entanglement. The solid lines are theoretical plots and the error bars represent one standard deviation.

We first examine if our entanglement distribution protocol via QT works well for amplitude damping decoherence. When each qubit undergoes independent amplitude damping decoherence quantified by *D*_1_ and *D*_2_, the amount of entanglement (concurrence) of the resulting two-qubit state is given by^12^


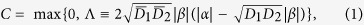


where 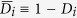 with *i* = 1, 2. The experimental results are shown in Fig. 3 and it shows clearly that, without QT, entanglement is degraded with increasing decoherence. In particular, Fig. 3(b) shows entanglement sudden death (which happens only if 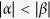) very clearly^24^,^25^. On the other hand, with QT applied before and after the decoherence channels, high-fidelity entanglement distribution is well demonstrated as the experimental data in Fig. 3 do not show any degradation of entanglement.

Let us now investigate if our entanglement distribution protocol via QT will work for a commonly-encountered form of decoherence in an optical fiber: polarization-mode dispersion (PMD)^26^. To more easily induce the effect of PMD, we use a set of quartz plates rather than using a long optical fiber, see Fig. 2(c). Due to the difference in refractive indices of 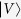 and 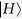 polarization in the quartz plates, the orthogonal polarization modes become separated in time and becoming distinguishable, causing degradation of entanglement. In experiment, PMD is increased by adding a set of anti-reflection coated quartz plates, one by one.

**Figure 4:**
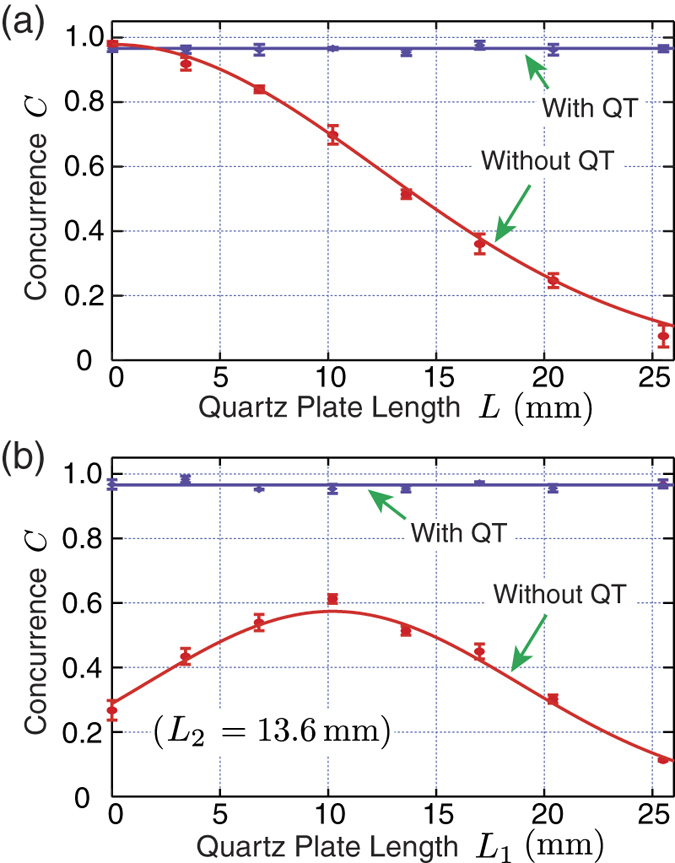
Experimental results: polarization-mode dispersion. The polarization-mode dispersion is introduced by inserting a series of quartz plate. Here, 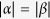 for both (**a**,**b**). In (**a**), both qubits experience the same decoherence (*L*_1_ = *L*_2_ = *L*). In (**b**), qubit 1 experiences the decoherence *L*_1_ while qubit 2 experiences a fixed decoherence *L*_2_ = 13.6 mm. The data show clearly that, without QT, concurrence is reduced, but with QT, concurrence remains the same. The solid lines are theoretical plots due to and the error bars represent one standard deviation.

It is clear from the experimental results shown in Fig. 4 that, without QT, decoherence caused by PMD reduces the amount of entanglement. However, when QT is applied before and after the PMD decoherence, concurrence is constant regardless of the amount of the PMD decoherence introduced.

## Discussion

Quantum coherence and entanglement, which are essential resources for quantum information, are often degraded and lost due to decoherence caused by unwanted and sometimes unavoidable system-environment interactions. Here, we have proposed and demonstrated high-fidelity entanglement distribution over decoherence channels via qubit transduction. By unitarily switching the initial qubit encoding to another, which is insensitive to particular forms of decoherence, we have demonstrated that it is possible to avoid the effect of decoherence completely.

In experiment, we have demonstrated high-fidelity distribution of photonic polarization entanglement over quantum channels with two types of decoherence in the polarization mode, amplitude damping and polarization-mode dispersion, via qubit transduction between polarization qubits and dual-rail qubits. For long-distance distribution, dual-rail qubits may require active phase-locking between the two spatial modes. Such active phase-locking can be achieved by sending classical locking signals along the optical paths^27^,^28^. Alternatively, qubit transduction between polarization qubits and time-bin qubits may also be used^29^.

We also note that our protocol is, unlike protocols relying on decoherence-free subspace, input-state independent, requires no ancillary photons and symmetries, and has near-unity success probability. With the development of better linear optics and high-speed electro-optic modulations, almost lossless qubit transduction among photonic qubits will be possible, enabling long-distance high-fidelity distribution of polarization entanglement for quantum information tasks. Moreover, qubit transduction technologies are expected to be essential for enabling quantum technologies with hybrid systems^30^.

## Methods

### State preparation

The initial two-qubit polarization-entangled state is prepared from photon pairs generated by spontaneous parametric down conversion process. We exploit 405 nm diode laser operating at 100 mW to pump a 6 mm thick type-I *β*-BaB_2_O_4_ crystal. A pair of 810 nm photons is generated on the frequency-degenerated, non-collinear phase matching condition. The down-converted photons are frequency-filtered by a set of interference filters with a full width at half-maximum bandwidth of 5 nm. Then, a pure two-qubit polarization entangled state 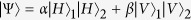 is prepared using a two-photon interference effect at a beam splitter^31^.

### Qubit transduction

The polarization qubit is unitarily switched to a dual-rail qubit, and vice versa, by using a polarization-dependent beam displacer (BD) and a 45° oriented half-wave plate (HWP)^20^,^21^ as shown in Fig. 2(a). The BD separates the two orthogonal polarization components, 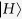 and 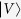, spatially by 4.0 mm at the output. After transmitting the BD, an arbitrary polarization qubit 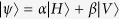 is converted to 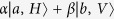. Then, after the 45° oriented HWP, 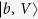 is transformed into 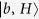 and we obtain 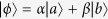. If QT is applied to the polarization entangled state 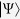, we get a two-qubit dual-rail entangled state 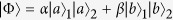. Note that QT is near deterministic between polarization and dual-rail qubits and polarization and time-bin qubits^20^,^21^,^22^,^23^ if optical elements are of high quality. The two-qubit quantum state is then analyzed by quantum state tomography^32^.

### Decoherence

We consider two types of decoherence on the polarization mode: amplitude damping shown in Fig. 2(b) and polarization-mode dispersion shown in Fig. 2(c). Under amplitude damping decoherence, a system qubit (S) interacts with the environment qubit (E) by the following map: 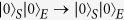, 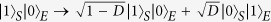, where *D* is the magnitude of the decoherence and 0 ≤ *D* ≤ 1^24^. In our experiment, the system qubit is the polarization qubit and the environment qubit is the path qubit shown in Fig. 2(b), and the amplitude damping decoherence for the polarization mode is realized with a partially polarizing beam splitter (PPBS) and a normal beam splitter (BS), such that the path length difference between the short and long paths is much bigger than the coherence time of the single-photon^12^,^13^,^24^. For the decoherence effects due to polarization-mode dispersion, we use a set of anti-reflection coated quartz plates such that the optic axis of the plates are cut parallel to the surface of the plates. Due to the birefringence, different group velocities of the two orthogonal polarization components lead to polarization-mode dispersion which causes dephasing decoherence once the temporal degree of freedom is traced out^26^.

## Additional Information

**How to cite this article**: Lim, H.-T. *et al.* Experimental demonstration of high fidelity entanglement distribution over decoherence channels via qubit transduction. *Sci. Rep.* **5**, 15384; doi: 10.1038/srep15384 (2015).
